# The Role of Artificial Intelligence in Cardiac Amyloidosis: A Focus on Diagnosis and Clinical Application

**DOI:** 10.3390/jcdd12060221

**Published:** 2025-06-11

**Authors:** Roshan Wardak, David Snipelisky

**Affiliations:** Cleveland Clinic Florida, Weston, FL 33331, USA

**Keywords:** artificial intelligence, machine learning, neural networks, deep learning, cardiomyopathy, cardiac amyloidosis

## Abstract

The aim of this review is to provide an update on contemporary and evolving artificial intelligence (AI) methods and their role in diagnosing and managing cardiac amyloidosis (CA). AI is the broadest term which describes a variety of different techniques that enable computers to mimic human intelligence. It is widely used across different diagnostic tests including electrocardiograms, echocardiography, scintigraphy and cardiac magnetic resonance (CMR). Through a comprehensive search among four databases, we identified several insights into clinical applications, diagnostic modalities and different utilization of AI in CA. The elusive nature of CA, which often makes early diagnosis challenging, can greatly benefit from the integration of AI into the diagnostic process. The variability in diagnostic strategies of CA underscores the need for more AI-focused prospective clinical trials to establish evidence-based guidelines for AI-driven diagnostic pathways. Our review highlights the capabilities of AI, particularly in the diagnosis of cardiac amyloidosis.

## 1. Introduction

Cardiac amyloidosis (CA) is caused by the accumulation of misfolded amyloid fibrils in the heart muscle and commonly progresses to heart failure, conduction system disease, and cardiac death. It is a serious and often fatal condition that is frequently misdiagnosed or undiagnosed until later in the disease course [1,2,3,4,5]. The two most common types of CA are light chain amyloid (AL) and transthyretin amyloid (ATTR), with ATTR-CA recently becoming more common in developed nations [1]. CA has been reported to be the etiology in up to 13% of patients with heart failure with preserved ejection fraction, and ATTR is present in approximately 16% of patients with severe calcified aortic stenosis undergoing transcatheter aortic valve replacement [6].

Unfortunately, most patients present with nonspecific symptoms and are often misdiagnosed as hypertrophic cardiomyopathy, ultimately delaying diagnosis and treatment of this progressive disease process. When screening for CA, it is important to recognize the following characteristics, including unexplained increase in left ventricular (LV) wall thickness (≥12 mm), specific echocardiographic findings and a multiparametric echocardiographic score [1]. Monoclonal protein assessment is also an essential step in the diagnosis of AL CA. The use of bone scintigraphy, including 99mTechnetium (Tc)-labeled tracers such as 3,3-diphosphono-1,2-propanodicarboxylic acid (DPD), hydroxymethylene diphosphonate (HMDP), and pyrophosphate (PYP), is an important non-invasive method frequently used for diagnosing ATTR-CA [1].

Due to the often challenging and late recognition of CA, artificial intelligence has emerged as a helpful diagnostic tool for early detection and disease management. AI encompasses many different areas of medicine, and the field of cardiac amyloidosis is no exception to that. The era of electronic health records, digital acquisition of data such as images, text and mapping data and the ability to store large datasets in the cloud has facilitated the growth of AI in CA [1]. Studies have shown use of AI in different modalities including ECG, echocardiography, scintigraphy, CMR, pathology and even serum studies.

Before we dive deeper into the use of AI in the field of CA, it is important to highlight the fundamental concepts of what AI is. It is usually an umbrella term referring to computational algorithms that attempt to imitate human behaviors to carry out tasks requiring human intelligence [2]. It contains subsets such as machine learning (ML) which involve computational, mathematical, and statistical algorithms that aim to learn directly from data without the necessity of explicit programming [2]. Furthermore, deep learning (DL) is an ML algorithm that consolidates all of these steps into a single package. DL algorithms can mimic human visual system behavior by presenting different data abstraction levels in their architecture [2].

The applications of AI are vast, including but not limited to imaging, diagnostics, treatment and prognosis, electrophysiology, interventional cardiology, and heart failure. The aim of this review is to introduce presently available AI-based techniques for the accurate diagnosis and treatment of CA and unlock their potential to change the way knowledge is generated, data is interpreted, and decisions are made [1].

## 2. Materials and Methods

**Figure 1:**
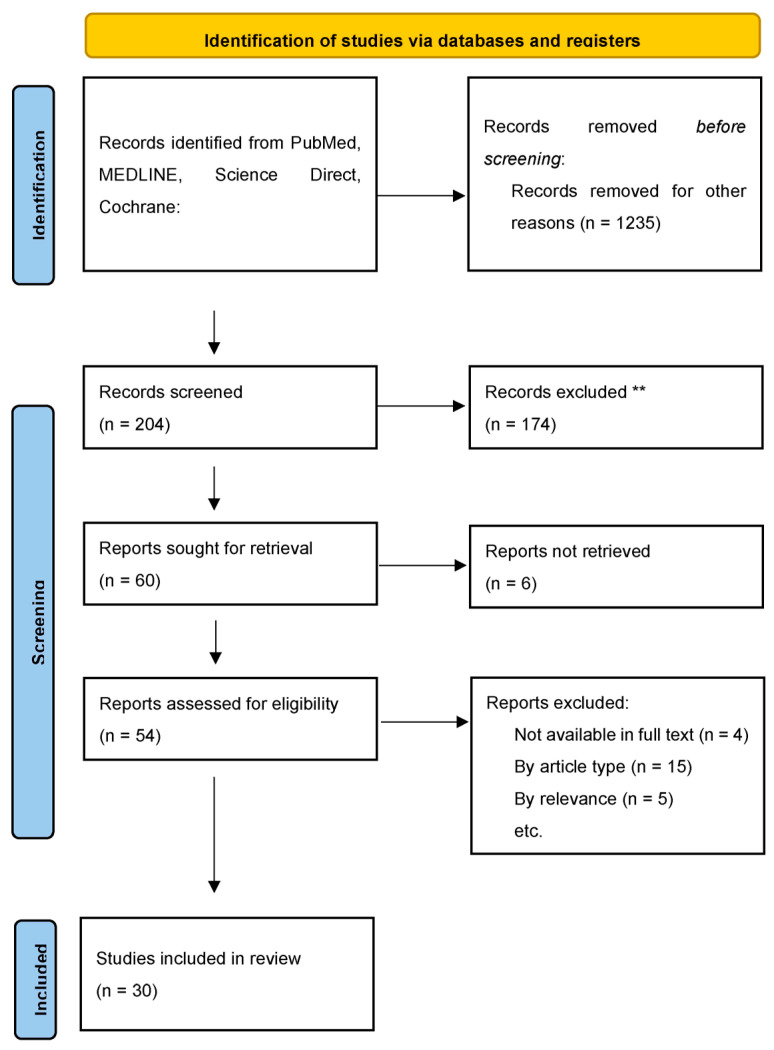
Identification of studies. ** Based on availability, article type, and relevance.

We conducted a systematic review of the literature using PubMed, MEDLINE, ScienceDirect, and the Cochrane Library to examine the clinical application of AI in cardiomyopathies (Figure 1).

The initial search identified 1439 records. After screening titles and abstracts, 1235 records were excluded. Of the 204 full-text articles assessed for eligibility, 174 were excluded based on availability, article type, and relevance. A total of 30 studies were included in the final review. Relevant references from these studies were also reviewed. Tables 1–4 includes pertinent studies further described.

The selection criteria and scope of the reviewed literature centered on studies demonstrating the effective application of AI in the diagnosis of CA. Given the heterogeneity of study design and outcomes, no meta-analysis was performed. The review adhered to PRISMA guidelines where applicable.

## 3. Discussion

### 3.1. A Brief Introduction to Machine Learning

Artificial intelligence is a computer system able to perform tasks that ordinarily require human intelligence to understand perceptions from the environment and perform actions using algorithms, heuristics, pattern matching, rules, deep learning, and cognitive computing [1]. It can create algorithms that can perform tasks to mimic human intelligence. These algorithms essentially “teach” computers to analyze vast datasets in a quick, accurate and efficient way with the use of complex computing and statistical algorithms [1]. The algorithms can identify patterns from new data that match with existing data from the algorithms they have already “learned from” to ultimately make predictions based on them [1].

Machine learning (ML) requires large amounts of data for it to educate itself through analysis resulting in the development of an algorithm, known as the training stage [1]. There are several factors that can affect the final performance of the model when it is being trained. However, the most important part of creating a machine learning model is the dataset used for training and testing the model [3].

Machine learning uses a novel toolset to shift from traditional statistical methods towards novel platforms for the integration and elaboration of clinical data. In doing so, it is revolutionizing medical research and ultimately paving pathways to better understanding and detection of cardiomyopathies [4].

### 3.2. A Brief Introduction to Neural Networks and Deep Learning

As mentioned previously, AI is often an umbrella term used interchangeably between machine learning, neural networks (NNs) and deep learning (DL). Although these terms are related, they have distinct meanings and cannot be used interchangeably. In efforts to simplify the concept, we can think of machine learning as a subset of AI and neural networks as a subset of machine learning. NNs are made of interconnected nodes or artificial neurons that work together to solve problems, mimicking how biological neurons signal to each other in the human brain [1]. Deep learning, on the other hand, is a subfield of machine learning. It can be thought of as an evolution of machine learning because it is made up of several layers of neural networks. This allows it to understand and mimic more complex and abstract behaviors. It allows for the processing of unstructured inputs such as images. Systems trained with DL are far better than humans at image recognition [1].

Basic ML uses algorithms to analyze data and learn from that data to make informed decisions based on what it has learned. While ML models do improve at performing specific functions as they take in new data, they still need human intervention. For example, if an ML algorithm returns an inaccurate prediction, an engineer must step in and make adjustments. Meanwhile DL structures algorithms in layers to create a network that can learn and make intelligent decisions on its own. It can learn through its own method of computing without the need of humans, which separates it from ML. Both ML and DA are modeled on the human brain, representing an even more sophisticated level of AI.

### 3.3. Using AI to Detect CA from Electrocardiogram

The electrocardiogram (ECG) is one of the most widely used diagnostic tests in the field of cardiology. It is fast, easily obtainable and often acquired in clinic visits. Table 1 lists pertinent clinical trials utilized the ECG in the detection of CA. Tison et al. developed an automated, scalable and interpretable method of performing detailed longitudinal tracking and comparison of ECGs [7]. They used a combination of machine learning methods including convolutional neural networks (CNNs) and hidden Markov models. After analyzing 36,186 ECGs they were able to train and interpret machine learning models to identify different pathologic cardiac structures and disease states with area under the receiver operating characteristic curve (AUROC) of 0.86 for cardiac amyloid.

Similarly, Goto et al. created an AI-ECG model for detecting CA with an AUC of 0.85 to 0.91 using ECGs capable of identifying CA before clinical diagnosis [1,5]. Grogan et al. developed an AI tool to detect CA from standard 12-lead ECGs, achieving an AUC of 0.91 and predicting CA over six months before clinical diagnosis in 59% of patients [5,8]. Harmon et al. performed internal validation of their AI-ECG for amyloidosis with an overall AUC of 0.84. Arana-Achaga et al. developed a prediction model named T-Amylo, which was the first of its kind, with an AUC of 0.92 for ATTR-CM using a combination of clinical, ECG, analytical and echocardiographic variables [12]. The best diagnostic accuracy was obtained from a combination of variables including age, male sex, previous carpal tunnel syndrome, interventricular septal thickness, and low voltages on ECGs. T-Amylo differs from other AI prediction models in that it includes carpal tunnel syndrome. It is a well-known prognostic marker in ATTR-CA and precedes the diagnosis of CA by 5 to 9 years, regardless of cardiac involvement [12]. The presence of carpal tunnel syndrome along with other ECG and echocardiographic findings make CA highly probable. This association presents high diagnostic accuracy and could be used for early diagnosis. The success of this prediction model comes from using these variables and the authors suggest it can be universally applicable once the suspicion of ATTR-CM is established.

Haimovich et al., used a pretrained CNN to develop an artificial intelligence-enabled ECG model named LVH-NET to detect and classify LVH and its associated disease states with an AUROC for cardiac amyloidosis of 0.95 [9].

Vrudhula et al., sought to train an AI model to identify CA from ECGs using different levels of case definitions, or variables representing less curated cases of amyloid to more defined cases of amyloid. In doing so, they hoped to understand whether centers without dedicated amyloid clinics would be able to train AI models using less stringent case definitions or criteria. Three progressively more stringent definitions of amyloid were used to train the model. The broadest case definition was a diagnosis of amyloid by International Classification of Diseases-9/10 (ICD-9/10) code. The second case definition was defined by a subset of patients from the first cohort but also having evidence of cardiac involvement which was defined by having an abnormal interventricular septum measurement and elevated brain natriuretic peptide or troponin. After ruling out AL, the third case of patients were patients who were seen in cardiac amyloid clinics with documented diagnosis by biopsy or 99mTc-technetium pyrophosphate scintigraphy (PYP) scan [6]. The models performed similarly in screening for CA, regardless of the selection of cases used to train the model. This data suggests that institutions without a dedicated amyloid clinic can still train meaningful models to identify CA from ECG, even if they use ICD 9/10 codes alone [6]. This idea can help bridge the gap for the diagnosis of rare diseases like CA. In order to understand models better and to incorporate them into clinical practice, we need prospective studies and multi-institutional validation [6].

The methodologies used in each of these studies varied, encompassing ML-, DL- and CNN-based algorithms. Despite differences in approach, these ECG based models shared a common objective and achieved an average AUC of 0.86. Performance ranged from the lowest AUC of 0.714–0.733 reported by Vrudhula et al., to the highest AUC of 0.95 reported by Haimovich et al. with LVH-Net and LVH-Net Leads I and II. The success of these algorithms, particularly LVH-Net, emphasizes the high accuracy in diagnosing cardiomyopathies and supports the consideration of screening for CA.

However, they have limitations, particularly considering that some studies are only single-center and other studies show poor AI-related performance with certain populations. Further research should aim to expand patient populations to include international healthcare settings.

### 3.4. AI Detected CA Using Echocardiography

Increased left ventricular (LV) wall thickness is a pathognomonic finding of CA in echocardiography, yet this can frequently be seen with other etiologies including hypertrophic cardiomyopathy (HCM) and hypertensive heart disease. Certain echocardiographic findings can help advance the work up of CA. Diastolic dysfunction grade 2 or more, decreased tissue doppler s’, e’, and a’ wave velocities (<5 cm/s), and reduction in LV global longitudinal strain (LGLS) < −15% [5] are clues to a possible patient with CA. Two of these three can help guide clinicians to making a diagnosis of CA (Table 2).

Zhang et al. used 14,035 echocardiograms over a 10-year period to train CNN models. He incorporated several tasks including automated identification of 23 viewpoints and segmentation of cardiac chambers across five common views. He was successfully able to quantify chamber volumes, left ventricular mass, determine ejection fraction and facilitate automated determination of longitudinal strain through speckle tracking [13]. They were able to successfully develop models to detect three disease states including HCM, CA, and pulmonary arterial hypertension.

Chao et al. developed ResNet50 which used a deep learning approach based on transthoracic echocardiography to differentiate constrictive pericarditis (CP) from restrictive disease, which is commonly seen among patients with CA. The deep learning model had a performance with AUC of 0.97 to differentiate CP from CA (based on restrictive physiology/disease) [14]. This study was limited by the small sample size of 381 patients.

Duffy et al. developed an Echonet-LVH model that can differentiate HCM and CA from other etiologies of LVH with CA AUC of 0.83. The first component was an end-to-end deep learning model to help determine left ventricle dimensions. The second component utilized a CNN to help predict the etiology of LVH [15]. It was effectively a deep learning system for quantifying left ventricular hypertrophy on echocardiography with automated prediction of LVH etiology [15]. The DL model was able to accurately identify subtle changes in LV wall geometric measurements and the causes of hypertrophy [15]. Li et al. used the first echocardiographic-based, automated deep learning model with a fusion architecture to differentiate the major etiologies of increased left ventricular wall thickness [16]. The final fusion model was able to identify CA with AUC of 0.90. Due to the underdiagnosed nature of CA and the fact that many centers do not routinely use LV strain imaging, the authors suggest that an AI-enabled, auto-populated reminder in the echocardiogram reading system can address this limitation and possibly facilitate earlier diagnosis [16].

Goto et al. developed a video-based echocardiography model for CA with good performance from five academic medical centers across two countries. It was able to successfully distinguish CA from disease states including HCM, HTN, and end-stage renal disease (ESRD). Furthermore, the authors had two expert cardiologists attempt to diagnose CA and compared these to the AI model results. In three of the five institutions, the model AUC outperformed the human readers and in the remaining two centers the result was within a 95% confidence interval [1].

The ability of data-driven machine learning to distinguish between different disease states has been shown to be quite impressive. It can accurately identify different cardiac structures, dimensions and, depending on the type of modality used, predict certain disease states such as CA. The rapid assessment by AI-generated models to determine and predict disease states makes it possible for clinicians to have real-time screening of cardiovascular diseases in a clinical setting. If this were to be used in real-world practice, it would substantially improve opportunities for quicker diagnosis and more targeted therapies.

### 3.5. Can AI Help Novice Operators Obtain Echocardiographic Images?

Echocardiography is one of the most commonly utilized cardiac imaging modalities and is usually performed by expertly trained sonographers and interpreted by board-certified cardiologists. However, there is a momentous shift toward using echocardiography in not just the aforementioned settings but also in everyday clinical settings using point-of-care ultrasound (POCUS). These usually involve clinicians and other medical personnel using handheld or portable machines without the training of sonographers and interpretation of board-certified cardiologists. POCUS is frequently used in settings like emergency departments, intensive care units, outpatient and preoperative clinics, and medically underserved areas [17]. It allows clinicians to quickly ascertain images to help guide their decision making and therapeutics. However, the quality of such exams and the risk of nondiagnostic or misleading imaging is there.

Narang et al., sought to answer the question of whether AI could help novice operators obtain echocardiographic scans. They developed a DL technology which was authorized by the US Food and Drug Administration (FDA) to provide real-time prescriptive guidance (turn-by-turn instructions) to novice operators to obtain transthoracic echocardiographic (TTE) images that allow for limited diagnostic assessment of cardiac chambers [17]. Eight nurses without prior ultrasonography experience used AI guidance to scan 30 patients each with a 10-view echocardiographic protocol. When compared to expert review, they were able to accurately identify left ventricular size and function in 98.8% of patients, right ventricular size in 92.5% and the presence of pericardial effusion in 98.8% [17]. This study highlighted another use of AI and its use in this setting can help increase the practice and yield of POCUS.

Similarly, Oikonomou et al. developed a CNN using a POCUS-adapted, multi-label, video-based model to differentiate between hypertrophic cardiomyopathy (HCM) and ATTR. The objective was to develop and test video-based deep learning algorithms for efficient diagnosis of HCM and ATTR [18]. The model was able to successfully discriminate HCM with an AUC of 0.903 in the first cohort and 0.890 in the second cohort, and ATTR with an AUC of 0.907 in the first cohort and 0.972 in the second cohort [18]. This study reinforces the confidence that challenging-to-diagnose diseases, such as HCM and CA, can be effectively identified through the use of AI adapted for POCUS.

The methodologies used in these studies primarily focused on deep learning algorithms. These echo-based models shared a common objective, and most models achieved an AUC of greater than 0.9 for detecting CA. Performance ranged from the lowest AUC of 0.83 for detecting CA reported by Duffy et al., to the highest AUC of 0.98 for detecting ATTR reported by Oikonomou et al.

However, significant limitations still exist for patients in remote regions with limited access to echocardiography. Soh et al. studied 79 patients in remote sites from referral centers and found most patients who were assessed with AI-echocardiography were found to have abnormal findings and less than half had management changes [20]. It suggests that better access to echocardiography in remote areas can improve diagnosis and management of cardiovascular disease.

With this notion, it is important to highlight that even if the AI model is not the most advanced at detecting different disease states, it can still be helpful to have a model which can predict the ejection fraction or identify heart failure with reduced ejection fraction. Ouyang et al. developed a video-based deep learning algorithm, EchoNet-Dynamic, that can segment the left ventricle, estimate the ejection fraction and assess for cardiomyopathy [19]. Their model was trained on echocardiogram videos and accurately segments the left ventricle with a Dice similarity coefficient of 0.92, predicts ejection fraction with a mean absolute error of 4.1% and reliably classifies heart failure with a reduced AUC of 0.97 [19]. Their conclusion is that by using information across multiple cardiac cycles, the model can rapidly identify subtle changes in ejection fraction, is more reproducible than human evaluation and can give a precise diagnosis in real time.

### 3.6. Scintigraphy Focused AI in Detecting CA

The use of technetium-99m (99mTc) whole-body bone scintigraphy (WBS) combined with gammopathy testing is a reliable and non-invasive diagnostic method for detecting CA [21]. The two main proteins deposited in the heart are TTR and light chains. Until 2016, myocardial biopsy was the only modality to reliably diagnose CA; however, it has associated risks [22]. Scintigraphy with amyloid-avid tracers has revolutionized the diagnostic pathway because intense cardiac uptake in the absence of pathologic light chains now enables a non-biopsy diagnosis of ATTR [22]. Although scintigraphy is diagnostic for ATTR, most scans are not ordered for this purpose. In fact, most scintigraphy scans are ordered for orthopedic and oncologic indications. This has led to several studies investigating scintigraphy databases in search of significant cardiac uptake in efforts to identify undiagnosed patients with CA. It is estimated that the prevalence of cardiac uptake grade ≥ 2 is between 0.3% and 3.45% [21]. Table 3 demonstrates pertinent trials utilizing AI in scintigraphy for the diagnosis of CA.

Delbarre et al. sought to develop and validate a deep learning-based model that automatically detects significant cardiac uptake (Perugini grade ≥ 2) on WBS from large hospital databases to assess patients at risk of CA [21]. They developed a CNN which was effective at identifying patients with cardiac uptake Perugini grade ≥ 2 on WBS and could help in the diagnosis of patients with CA [21]. This study, however, was limited by the fact that it was based solely on Perugini score and that, although a high score is highly suggestive of CA, there are a number of cases that are not related to amyloidosis.

Halme et al. also trained a CNN to automatically detect and classify ATTR from scintigraphy images using two custom-made CNN architectures to discriminate between the four Perugini grades of cardiac uptake and compare them to four state-of-the-art CNN models. They found that their models performed better than, or equally as well as, the state-of-the-art CNN models the in detection and classification of cardiac uptake [23]. This suggests that automated CNN models can be trained to accurately detect and classify different grades of cardiac uptake on bone scintigraphy.

Spielvogel et al. developed the largest study to date for the development and validation of an automated system for the detection of CA using 16,241 patients and 19,401 scintigraphy scans from nine centers internationally. After validation, their AUC across the different cohorts were between 0.925 and 1.000 [22]. The AI’s predictions showed statistically significant prognostic value for overall mortality.

The methods used in these studies primarily focused on deep learning algorithms. These scintigraphy-based models shared a common objective, and achieved a high AUC for detecting CA. Performance ranged from the lowest AUC of 0.88–0.94 for detecting ATTR reported by Halme et al., to the highest AUC of 0.99 for detecting cardiac uptake Perugini ≥ 2 reported by Delbarre et al. This further underscores the utility of AI-based screening of cardiac amyloidosis in patients undergoing scintigraphy. It can be a reliable way of identifying potential patients with amyloidosis which may have otherwise been missing or difficult to diagnose.

### 3.7. Use of CMR to Aide in the Diagnosis of CA

CMR plays an important role in providing highly accurate and detailed characterization of cardiac tissue and morphology which can distinguish between CA and other cardiac disease states. With a non-invasive approach, it can play a decisive role in the diagnosis of both ATTR and AL CA subtypes. The primary imaging findings are derived from the late gadolinium enhancement (LGE) pattern, T1 mapping, and extracellular volume (ECV). Although the values for ECV are not specific, consensus in the literature suggests that ECV > 40% are highly suggestive of CA [24].

There are several CMR findings that can be quite specific for CA, particularly a pattern of variable biventricular pseudohypertrophy with diffuse subendocardial-to-transmural late gadolinium enhancement [25]. In addition to both diffuse transmural and subendocardial LGE, abnormal gadolinium kinetics must also be present [5]. Subendocardial LGE is more prevalent in AL CA while transmural LGE is more prevalent in ATTR CA [24]. Due to an exceedingly variable appearance of CA on CMR across different stages, interpretation can be highly dependent on operator experience. Table 4 represents pertinent studies evaluating such with AI mechanisms. 

Martini et al. developed a DL approach to diagnosing CA which was compared to an ML-based approach that simulated CMR reading by experienced operators. The two main advantages of DL are speed and accuracy. There are several limitations to this study, of which some included a small sample size, a high prevalence of CA, lack of external validation and use of ML as a surrogate for expert reading. However, the importance of this study demonstrates the feasibility to implement a DL algorithm to provide support to CMR reading when patients are referred for suspected CA [25].

Agibetov et al., using CMR, developed a CNN algorithm for the diagnosis of CA. They were able to successfully achieve an average ROC AUC score of 0.96. This resulted in 94% sensitivity and 90% specificity. They were able to demonstrate that there is a potential to establish a fully computational diagnostic path for the diagnosis of CA [26].

Recent investigations have shown that cardiac strain provides discriminative value for all cardiac chambers and thus may support differentiation of CA from HCM and healthy control subjects [27]. Eckstein et al. sought to answer the question of whether a high CA diagnostic accuracy was achievable using strain measurements. This study used a support vector machine (SVM) algorithm comparing the performance of CMR in CA patients to corresponding data from HCM patients and healthy control subjects. They were able to conclude that SVM accurately differentiates CA from a cohort of HCM patients and healthy control subjects. These observations have the potential to make non-contrast CMR an important part of clinical decision making [27].

The methods used in these studies primarily focused on deep learning algorithms. These CMR-based models shared a common objective, and on average achieved the highest AUC for detecting CA compared to ECG, echocardiography, and scintigraphy studies. Performance ranged from the lowest AUC of 0.96 for detecting CA reported by Agibetov et al. and Eckstein et al., to the highest AUC of 0.98 for detecting CA reported by Martini et al. These studies highlight the remarkable advancements in the performance of AI models in identifying CA during CMR. Although there are limitations within these studies, the future holds promise for AI becoming an increasingly integrated component of the diagnostic work-up for CA.

### 3.8. Differentiating CA from Other Cardiac Disease States

One of the key challenges in diagnosing CA is its potential to remain undetected or unrecognized for years, often until clinical symptoms of heart failure emerge. We evaluated different AI modalities and their rates of detection of various cardiac diseases, some of which may mimic the presentation of CA. Tison et al., developed a machine learning approach to ECG interpretation to detect four different disease states including pulmonary arterial hypertension (PAH), HCM, CA, and mitral valve prolapse (MVP) [7]. After first training a CNN-based model, they then trained a multilayered neural network to detect different segments within an ECG. In addition to that, they used a Gradient Boosted Machine (GBM), which is machine learning algorithm with an ensemble regression-tree-based technique. These were further trained and validated 5-fold. The model was able to detect PAH the strongest with an AUROC of 0.94, followed by HCM with an AUROC 0.91, CA with AUROC of 0.86 and MVP with the weakest discrimination with an AUROC of 0.77 [7].

In a similar study, Goto et al. developed machine learning models to detect HCM and differentiate it from other cardiac conditions using ECGs and echocardiograms. After the model was trained in a federated manner using ECGs, it was able to discriminate HCM from hypertension, aortic stenosis, and CA with AUROC of 0.84, 0.83, and 0.88, respectfully [10]. The echocardiogram model was able to discriminate HCM from hypertension, aortic stenosis and CA with AUROC of 0.93, 0.94, and 0.85, respectively.

AI models can vary depending on their training, which is particularly important when attempting to differentiate between multiple disease states. Haimovich et al. developed an ECG algorithm, LVH-Net, to discriminate between different LVH etiologies. It was successful in detecting and discriminating between CA, HCM, AS, hypertensive LVH and other LVH etiologies [9].

LV wall thickness is a common finding in echocardiography with various etiologies, including HCM, CA and other conditions such as hypertensive heart disease. It is important to quickly and accurately identify the etiology in order to treat the underlying disease process. Li et al. developed an automated deep learning model with a fusion architecture to facilitate the evaluation and diagnosis of LV wall thickness using echocardiography. The final fusion-based model outperformed all the view-dependent models with AUROC of HCM 0.93, CA 0.90 and other diseases including hypertension 0.92 [16]. This study set the stage for demonstrating a superior echocardiographic-based fusion model especially for CA patients without any segmentation of the images. This reinforces the idea that AI algorithms are capable of detection and classification of different cardiac disease states with remarkable precision and accuracy.

### 3.9. Use of AI in Genetics to Predict CA

AI is being used in several different areas of genetics in predicting the onset of disease states. One such application includes the use of machine learning in predicting pathogenic immunoglobulin light chain (LC) toxicity in light chain amyloidosis. Garofalo et al. developed LICTOR which is a machine learning approach to identifying somatic mutations during clonal selection. It was able to achieve a specificity and sensitivity of 0.82 and 0.76, respectively, with AUC of 0.87. This method showed that it can accurately predict LC toxicity, allowing for a timely identification of high-risk patients who would likely progress to AL [28]. It is an exciting advancement of AI with potential applications for other disease states including cancer.

### 3.10. A Hopeful Future of Diagnosing CA

Artificial intelligence has the potential to transform every facet of cardiovascular practice and research [29]. The rise in technology has enabled us to expand access to cardiovascular screening and monitoring, especially in areas where specialized care was historically difficult to obtain. With the integration of AI-enabled technologies into cardiovascular practice and investigation, we are now more than ever able to provide individualized diagnostic and therapeutic options to our patients. It enables a more personalized, precise and effective approach to caring for patients in the future.

This is particularly evident in ATTR CM, especially in relation to imaging. Several tasks can be performed by ML algorithms, including detection, classification, clustering, segmentation, regression, time-to-event prediction, image-to-image translation, and image captioning [2]. By combining clinical data with imaging data, we can expect improved diagnostic outcomes which may lead to early treatment and improved overall outcomes.

## 4. Limitations

Data are an essential component of any AI algorithm. In broadest terms, AI is the use of data to train models and enable them to make decisions and perform specific tasks. These data can consist of facts or information in different forms like sound, text, values and, importantly in our review, images. Proper interpretation of data is crucial for training models. Several parameters need to be defined in order to effectively train a model. These can include several parts such as the complexity of the model, the complexity of the learning algorithm, the need for labeling, the definition of an acceptable margin of error and the diversity of the inputs that are being used. The input data of a model needs to be prepared in terms of the task and the expected results. It can then proceed to data processing, which is beyond the scope of this review.

In these studies, specific datasets were used to train the AI models. Consequently, they must be interpreted in the context of each study’s limitations. Since their data was largely historical, having been chosen from a selection of certain patient populations in certain healthcare systems, it makes it difficult to predict the generalizability for all individuals. For example, if the study population was largely composed of older white individuals, then the results of the model may not generalize to younger individuals or those with varying racial or ethnic compositions. One way the authors have addressed this is by ensuring their AI models undergo validation. Another method that can be utilized is model retraining, which is a more intricate process that involves updating an existing model using a new dataset. This phenomenon is complex and was not used by the authors in these studies.

While it is reasonable to assume that these studies encountered various challenges, our review found that the authors did not explicitly address architectural limitations in relation to the specific complexities of cardiac imaging or ECG signal analysis. However, they did provide insight into their data acquisition strategies, occasionally employing innovative techniques.

Our retrospective review is inherently limited by potential biases, including selection and publication bias. The variability in AI models across the data we analyzed may affect our conclusions. Additionally, our findings depend on the accuracy and completeness of the data in these reports. Our insights into the diagnostic challenges and successes for AI-driven diagnosis of CA are drawn from retrospective data, underscoring the need for validation by prospective clinical trials. Additionally, there is no data assessing how AI will evolve as models “learn” from themselves over time.

## 5. Conclusions

The integration of AI into cardiology has transformed the field and has become an integral part of research and clinical practice. It has significantly improved the diagnosis of cardiomyopathies over recent years. However, certain conditions like CA are still underdiagnosed and a high clinical suspicion is generally needed to make the diagnosis.

This comprehensive review has highlighted the diverse applications of AI into CA, focusing on diagnostic innovations spanning from ECG, echocardiography, scintigraphy and CMR. These AI-driven technologies have demonstrated the potential to revolutionize traditional approaches to diagnosing CA. Enhanced imaging and interpretation have been a significant byproduct of advanced AI technologies, helping clinicians and patients alike.

There can be considerable variability in the ability of experts to detect CA. However, with AI-powered algorithms, the diagnostic landscape for detecting CA has undergone a paradigm shift enhancing accuracy and efficiency in interpreting various diagnostic studies. This shift will enable the emergence of precision medicine, leveraging AI to tailor treatment plans based on individual patient characteristics, genetic profiles and disease-specific details.

Notably, AI in the medical field is still at a rudimentary level. However, the full potential of AI is continuously being explored. The reality is that AI has come to stay and its future in healthcare is promising. The performance of AI algorithms expressed by their high AUC values are a demonstration of their efficiency and capability in achieving medical diagnosis. Their practicality lies in providing healthcare providers with a more innovative guide to enhanced decision making. They are not meant to be replacing physicians but rather to augment their practice to improve diagnostic accuracy, efficiency and an overall improvement in patient care.

Nonetheless, alongside these promising advancements, challenges regarding AI do exist. One such challenge is algorithm accuracy, since AI algorithms are created from collected data. There are several limitations when it comes to data, including but not limited to diversity, uniformity and availability. Interoperability is another challenge that can arise from AI algorithms. Due to the nature of the algorithms’ training, testing and validation, their use in other healthcare systems would have to be tested prior to implementation. Ethical considerations and integration into clinical workflows therefore make it a challenge to be able to use an AI algorithm widely [30]. The successful integration of AI into the field of cardiology requires careful consideration of these challenges. It requires ongoing research, validation, and strategic solutions, but the emphasis should be on removing barriers to translating AI in research to clinical medicine.

AI will continue to evolve and so must the roles of healthcare providers alongside it. With the many studies that have been undertaken in recent years, I believe AI-driven detection of CA is now feasible. However, it is important for clinicians and researchers to continue to collaborate closely in addressing the fundamental scientific challenges that underpin the effective integration of AI into clinical practice. Kyung-Hee et al. stress in their review of AI applied to cardiomyopathies the importance of ever-growing clinical research to ascertain the clinical utility of AI tools to help improve diagnosis and outcomes in cardiomyopathies. Similarly, Ahammed et al. emphasize the need for optimization of AI applications in clinical practice for improving patient outcomes. We stand at the dawn of a new era in AI-driven healthcare, and this review underscores the dedication and perseverance of the early pioneers who successfully harnessed AI to diagnose CA. It is imperative that we build upon their foundational work to advance the seamless integration of AI into routine clinical practice.

## Figures and Tables

**Table 1:** Electrocardiogram Studies.

Study	Year	Design	Sample Size	Population/Data	Objective	Key Findings	Limitations	Comparison to Other Disease States
Vrudhula et al. [6]	2022	Deep learning models	341,989	Cedars-Sinai Medical Center	Training AI models using different selection of cases and controls in screening for CA	AUC of 0.714–0.733 for detecting CA, similar performance using different curated cases for training	Model was not externally validated	N/A
Tison et al. [7]	2017	Machine learning algorithms	36,186	University of California, San Franciso (UCSF) ECG Database	Detect and track four disease states including PAH, HCM, CA, and MVP	AUC of 0.86 for predicting CA	Single center. Only optimized to analyze ECGs in normal sinus rhythm.	N/A
Goto et al. [1]	2021	CNN based model	10,933	Brigham and Woman’s Hospital System	Automated strategy to augment CA detection	AUC of 0.85–0.91 for detecting CA before clinical diagnosis	Probability of undetected cases in the control group, false labels	N/A
Grogan et al. [8]	2021	DNN algorithm	2541	Mayo Clinic	Detect CA from a standard 12-lead ECG	AUC of 0.91 for predicting CA more than six months before clinical diagnosis	Single center, uncertainty regarding cardiac involvement in some patients	N/A
Haimovich et al. [9]	2019	Deep learning models LVH-Net and LVH-Net Leads I and II	50,709	Mass General Brigham Healthcare System	Deep learning models to classify LVH etiology using 12-lead and single-lead ECGs	AUC of 0.95 for diagnosing CA	Patients with multiple LVH etiologies may be misclassified. Dataset included mostly older white individuals making it difficult to generalize to patients who are younger or those with different racial backgrounds	AUC for HCM 0.92; AUC for AS 0.90; AUC for HTN 0.76; AUC for other LVH 0.69
Goto et al. [10]	2021	Federated learning, a machine learning technique	56,129	Three US, one Japanese academic medical centers	ML models to detect and discriminate HCM from other causes of LVH using ECGs	AUC of 0.88 for discriminating CA	Features of the training model remain obscure resulting in some ambiguity	AUC for HCM 0.90–0.96; AUC for HTN 0.84; AUC for AS 0.83
Harmon et al. [11]	2022	Follow up validation study of AI-ECG DNN algorithm	7040	Mayo Clinic	Evaluate post development performance of the AI-enhanced ECG to detect CA with respect to different subgroups	AUC of 0.84, acceptable performance across various subgroups	Lower performance noted in LBBB, LVH, and ethnically diverse populations like Hispanics	N/A

**Table 2:** Echocardiogram Studies.

Study	Year	Design	Sample Size	Population/Data	Objective	Key Findings	Limitations	Comparison to Other Disease States
Goto et al. [1]	2021	CNN based model	4565	Three US, one Japanese academic medical centers	Automated strategy to augment CA detection	AUC of 0.89–1 for detecting CA before clinical diagnosis	Probability of undetected cases in the control group, false labels	AUC for HCM 0.87–0.96; AUC for HTN 0.89–0.96; AUC for ESRD 0.90–0.96
Zhang et al. [13]	2018	CNN based models	14,035 echocardiograms	UCSF	CNN to develop an analytic pipeline for the automated analysis of echocardiograms	AUC of 0.87 for detecting CA	Problems with segmentation arose due to complexity of tasks	AUC for HCM 0.93; AUC for PAH 0.85
Chao et al. [14]	2021	ResNet50 a deep learning model	381	Mayo Clinic	Deep learning approach based on echocardiography to differentiate CP from CA as a representative of restrictive cardiomyopathy	AUC 0.97 in differentiating CP and CA	Small sample size and single center. Designed to differentiate CP from CA but not necessarily other forms of RCM.	N/A
Duffy et al. [15]	2020	Deep learning algorithm	23,745	Stanford and Ceders-Sinai medical centers	Deep learning algorithm measuring LV dimensions and identifying HCM and CA	AUC of 0.83 for detecting CA	Certain populations with known prevalence of CA like Black individuals were underrepresented	AUC for HCM 0.98; AUC for AS 0.89
Li et al. [16]	2019	DL model with a fusion architecture	586	Mayo Clinic	Using DL model with fusion architecture to facilitate the evaluation and diagnosis of LV wall thickness	AUC of 0.90 for detecting CA	Single center, retrospective, potential referral bias	AUC for HCM 0.93; AUC for HTN/Other 0.92
Narang et al. [17]	2019	Deep learning algorithm	240	Northwestern Memorial Hospital and Minneapolis Heart Institute	Test whether novice users could obtain 10-view echocardiographic studies using DL-based software	Novice users identified LV size, LV function, pericardial effusion 98.8% of the time and RV size 92.5% using DL algorithm	Small sample size, no control group for novice scanners	N/A
Oikonomou et al. [18]	2024	Video-based deep learning algorithms	38,751	Yale–New Haven and Mount Sinai Health System	Develop video-based deep learning algorithms to diagnose HCM and ATTR on POCUS	AUC of 0.98 for detecting ATTR	Retrospective study. POCUS was not used to train the AI models	AUC for HCM 0.95
Ouyang et al. [19]	2018	EchoNet-Dynamic a video-based deep learning algorithm	10,030	Stanford Health Care	DL approach to segment the LV, estimate EF and assess for cardiomyopathy	AUC of 0.97 for predicting EF < 50%	Model was trained using videos obtained by trained sonographers	N/A
Goto et al. [10]	2021	Federated learning, a machine learning technique	6825	Three US, one Japanese academic medical centers	ML models to detect and discriminate HCM from other causes of LVH using echocardiograms	AUC of 0.85 for discriminating CA	Features of the training model remain obscure resulting in some ambiguity	AUC for HCM 0.90–0.96; AUC for HTN 0.93; AUC for AS 0.94

**Table 3:** Scintigraphy Studies.

Study	Year	Design	Sample Size	Population/Data	Objective	Key Findings	Limitations
Delbarre et al. [21]	2022	Deep learning algorithm	4681	Two Health Systems in France	Develop deep learning algorithm to detect significant cardiac uptake on scintigraphy to identify patients at risk of CA	AUC of 0.99 for detecting cardiac uptake Perugini grade ≥ 2	Model is solely based on Perugini score; therefore, unable to distinguish between amyloidosis-related and nonamyloidosis-related cardiac uptake
Spielvogel et al. [22]	2023	Deep learning algorithm	16,241	Nine International Centers	Develop deep learning algorithm to detect high-grade cardiac uptake on scintigraphy	AUC of 0.925–1 for detecting CA-suggestive uptake on scintigraphy	Study was based on visual image assessment of Perugini grade ≥ 2, suggesting CA
Halme et al. [23]	2021	CNN	1334	Four Finnish Centers	Train CNN to detect ATTR from scintigraphy	AUC of 0.88–0.94 for detecting ATTR patients	Small sample size of ATTR positive patients, simple architectures of CNN models, lack of clinical validation

**Table 4:** Cardiac Magnetic Resonance Studies.

Study	Year	Design	Sample Size	Population/Data	Objective	Key Findings	Limitations
Martini et al. [25]	2019	Deep learning and Machine learning algorithms	206	Italy	Assess diagnostic performance of CMR-based DL and ML algorithms	AUC of 0.98 for DL detection of CA; AUC of 0.95 for ML detection of CA	Small sample size, high prevalence of CA from a CA specialized center, no external validation
Agibetov et al. [26]	2018	CNNs	502	Austria	CNNs to recognize imaging patterns associated with CA	AUC of 0.96 for detecting CA, 94% sensitivity, 90% specificity	Single center, most patients had advanced HF, and my not identify early disease
Eckstein et al. [27]	2021	Machine learning algorithms	107	Germany	ML algorithms using multi-chamber strain and cardiac function	AUC of 0.96 for detecting CA	Small sample size, unmatched retrospective cohort, unsupervised algorithmic model

## Data Availability

No new data were created or analyzed in this study.

## Abbreviations

The following abbreviations are used in this manuscript:

AI	Artificial intelligence
AL	Light chain amyloid
ATTR	Transthyretin amyloid
CA	Cardiac amyloidosis
CMR	Cardiac magnetic resonance
ML	Machine learning
DL	Deep learning
NN	Neural networks
LV	Left ventricular
CP	Constrictive pericarditis
DPD	3,3-diphosphono-1,2-propanodicarboxylic acid
HMDP	hydroxymethylene diphosphonate
PYP	pyrophosphate
SPECT	single-photon emission computed tomography
CNN	convolutional neural networks
AUROC	area under the receiver operating characteristic curve
HCM	hypertrophic cardiomyopathy
LGLS	LV global longitudinal strain
TAPSE	tricuspid annular plane systolic excursion
ESRD	end-stage renal disease
POCUS	point-of-care ultrasound
FDA	US Food and Drug Administration
TTE	transthoracic echocardiographic
WBS	whole body bone scintigraphy
99mTc	technetium-99m
LGE	late gadolinium enhancement
ECV	extracellular volume
SVM	support vector machine
PAH	Pulmonary arterial hypertension
MVP	Mitral valve prolapse
GBM	Gradient Boosted Machine
LVH	Left ventricular hypertrophy
HTN	Hypertension
LC	Light chains
